# “Hey, We Can Do This Together”: Findings from an Evaluation of a Multi-sectoral Community Coalition

**DOI:** 10.1007/s11524-020-00473-3

**Published:** 2020-08-17

**Authors:** Lindsey Realmuto, Linda Weiss, Patrick Masseo, Kumbie Madondo, Rashi Kumar, Susan Beane, José A. Pagán

**Affiliations:** 1 College of Urban Planning and Policy, University of Illinois at Chicago, Chicago, IL USA; 2 Center for Evaluation and Applied Research, The New York Academy of Medicine, New York, NY USA; 3 BronxCare at the time of the study, currently NYC Deputy Mayor’s Office for Health and Human Services, New York, NY USA; 4 Healthfirst, New York, NY USA; 5 Department of Public Health Policy and Management, School of Global Public Health, New York University, New York, NY USA

**Keywords:** Multi-sectoral coalitions, health inequity, program evaluation, community building

## Abstract

Multi-sectoral coalitions focused on systemic health inequities are commonly promoted as important mechanisms to facilitate changes with lasting impacts on population health. However, the development and implementation of such initiatives present significant challenges, and evaluation results are commonly inconclusive. In an effort to add to the evidence base, we conducted a mixed-methods evaluation of the Claremont Healthy Village Initiative, a multi-sectoral partnership based in the Bronx, New York City. At an organizational level, there were positive outcomes with respect to expanded services, increased access to resources for programs, improved linkages, better coordination, and empowerment of local leaders—all consistent with a systemic, community building approach to change. Direct impacts on community members were more difficult to assess: perceived access to health and other services improved, while community violence and poor sanitation, which were also priorities for community members, remained important challenges. Findings suggest significant progress, as well as continued need.

## Introduction

In low-income communities that have experienced decades of disinvestment from the public and business sectors, interventions with a limited focus on individual behaviors and health conditions—and with a short time horizon—are unlikely to have substantial impacts on population health or its determinants [1, 2]. Broader initiatives [3], often including multi-sectoral coalitions focused on systemic challenges, are commonly considered to be important mechanisms to facilitate changes that are sensitive to local priorities [4] and are more likely to have widespread and lasting impact [5–7]. However, the development and implementation of such initiatives present significant challenges, including difficulty engaging all the necessary stakeholders, insufficient resources and/or time for systemic change, and limited political power [6]. Evaluation is also challenging given the scope and fluidity of multi-sectoral initiatives, difficulties delineating the affected population and defining an appropriate comparison group, the length of time needed to observe a relevant and measurable impact, and concurrent programs and initiatives that may be effectively working toward similar goals [6, 8]. Results from coalition evaluations are often inconclusive, and information on effective mechanisms demonstrating “value added” is still lacking [6, 7].

In an effort to add to the evidence base regarding multi-sectoral collaborations, we conducted mixed-methods evaluation of the Claremont Healthy Village Initiative (CHVI), which is a coalition based in Claremont, a section in the Morrissania neighborhood of the Bronx, New York City (NYC). Claremont is a low-income community with disproportionately high rates of poor health and premature mortality: 31% of Claremont residents live in poverty, 16% are unemployed, and 36% do not have a high school degree. Twenty-two percent of adults in Claremont have diabetes and 36% are obese. At 76.2 years, life expectancy is 5 years shorter than the NYC average [9].

Founded in 2012, CHVI is a partnership that brings together residents and multi-sectoral institutions to integrate programs and services to address the broader determinants of health, improve quality of life, create synergies that promote health and reduce inequities, and foster collaboration. Anchored by BronxCare, which is a local hospital, and Healthfirst, a not-for-profit managed care organization (MCO), partners include local tenant associations, community and senior centers, civic groups, public schools, arts and cultural organizations, sports and recreation groups, and NYC governmental agencies. These organizations collaborate on the design and delivery of programs, outreach to community members, and implementation of service activities [10].

CHVI is not a legal entity and lacks a hierarchical structure for decision-making. Rather, CHVI encompasses and supports a broad range of programs and activities developed and/or promoted by community stakeholders, including health fairs, exercise classes, a youth leadership council, anti-violence events, neighborhood clean-ups, cooking classes, health education (e.g., mental health first aid), and programs in the visual arts. CHVI hosts regular stakeholder meetings to facilitate collaboration and the exchange of information. CHVI also publishes a newsletter for community members that include information on community programs and activities; “spotlights,” describing local organizations and individuals; general announcements; and a job listing.

## Evaluation Methods

The evaluation, which was conducted from 2016 to 2018, used a mixed-methods approach to assess changes at the individual, organizational, and community level. It included interviews with staff from the two anchor institutions and key partnering organizations (referred to jointly in this study as “stakeholders”), focus groups and surveys with community members, a survey of CHVI partner organizations, and an analysis of health insurance claims data. The findings reported here focus on community perceptions of program effects, and, as such, the results rely on data from the interviews, focus groups, and community member surveys only. The protocol was approved by the Institutional Review Board of The New York Academy of Medicine.

### Key Stakeholder Interviews and Community Member Focus Groups

Stakeholder interviews were conducted at the start (*n* = 25) and end (*n* = 6) of the evaluation. Interviewees represented a range of institutions and individuals working in Claremont, including the MCO and hospital that founded the initiative; a senior center; a community center; tenant associations; NYC government agencies; a philanthropic organization; health care providers; and CBOs focused on youth, physical activity, healthy food access, and the arts. Interviewees were identified by the CHVI project leads, by the interviewees themselves, and through evaluator attendance at CHVI project meetings and activities. Both rounds of interviews included questions on engagement in and perceptions of CHVI, CHVI impact on community members and institutions from the time the initiative was launched, strengths and challenges, and recommendations for the future. The second-round interviews focused on changes occurring in the prior 2 years, since the start of the evaluation. Interviews were conducted in person using a semi-structured guide and were approximately 1 h.

A total of seven focus groups were conducted with Claremont residents (*n* = 94), including two comprised of youth and young adults, exclusively, and one with older adults. Participants were primarily female (65%), Black (80%) or Latinx (15%), and residents of public housing (62%). Ages ranged from 14 to 65 (mean = 49). Focus groups used semi-structured guides, including questions on health concerns in the community, health care access, community resources, and CHVI engagement. They were 60 to 90 min in length, and participants received $25 in cash.

All focus groups and interviews were audio-recorded and professionally transcribed. Data were maintained and coded for pre-identified and emergent themes using NVivo, a software package for qualitative analysis.

### Community Member Survey

An in-person community member survey was conducted over nine visits to Claremont between May 2017 and July 2018. The survey included questions on demographics; resident awareness and engagement with CHVI; health and health behaviors; community characteristics and services; perceptions of trust within the Claremont community; and community change over the prior 3 years, which—according to the CHVI leads—was the timeframe for the launch of potentially observable community programming. Questions on trust were adapted from the Integrated Questionnaire for the Measurement of Social Capital [11]. A transit card, worth roughly $10, was offered as an incentive for survey completion. The survey was available and administered in English and Spanish. Surveys (*n* = 162) were completed at community events and outside a neighborhood center. Eligibility was limited to Claremont residents (as determined by zip code), age 18 and older. The majority of participants were female (55%), Black (63%) or Latinx (34%), and residents of public housing (69%). Ages ranged from 18 to 83 (mean = 48). Survey data were analyzed using IBM SPSS software (version 19).

## Results

Below, we describe stakeholder and community member perspectives on CHVI impacts. We focus first on organizational impacts, including changes in community programming and resource availability. Subsequently, we describe perspectives on outcomes specific to the health and well-being of individuals living in Claremont.

### Impact on Community Programming

#### Stakeholder Perspectives

Participants in key stakeholder interviews described the CHVI collaborative process, which included regular meetings open to all partners, outreach and engagement by individual staff, as well as ongoing and consistent communication resulting in the development of sustainable working relationships. An important component and strength of CHVI, as described by stakeholders, was the involvement of community members in the process, as well as the elevation and cooperation of local Claremont leaders, most notably tenant association presidents.

I think we have really good communication. … my contact over at [the hospital], and we're in probably weekly conversation to make sure. And I think also that it's ongoing, you know, in the goals to make something sustainable. (Stakeholder interview)

[Community members] have been very involved, you know. All the people I work with, we call each other, “I’m having this, this, this. Come support me. Help me.” We all know who our leaders are, and also we trying to get more people to come on board to become leaders. (Stakeholder Interview)

I would say [a strength] is the involvement that they've gotten from leaders in the community. Like the tenants' association presidents and stuff. That's no small feat. (Stakeholder interview)

Stakeholders also described the variety of CHVI-affiliated programs and services their organizations offered, as well as the perceived benefit to community members, including increased knowledge and awareness and improved access to activities that promote health and wellness.

I think that you've got to realize … that conversations about healthy eating and exercise don't exist in the South Bronx. I've lived here for years and I've never even heard a conversation like that. So, the fact that they're introducing this, that they're just getting people thinking about it. That they are doing events around it. I think there is some ripple effect. (Stakeholder interviewee)

The Urban Ambassadors, so here, that has been successful, has had an impact. And not only have we had, let’s say, sixty kids go through leadership development, it’s also been a lever to affect kind of the priorities of those organizations and of the neighborhood, so introducing the idea about healthy living, right your path, getting people to think about their careers… so those types of things. (Stakeholder interview)

It's good because the community – there's so much going on and you don't expect one event or one thing to satisfy all the community. Whether it's youth service or children's services, something for seniors or something for adults, something that's inter-generational. We have mental health training coming up. There's a lot a moving parts, you wanna get folks out and you don't wanna burn people out. (Stakeholder interview)

Several stakeholders attributed their own organizational and program expansion to engagement with CHVI, describing increases in referral networks and linkages to health care services; financial and/or in-kind support provided by the anchor organizations; and connections to outside resources, including funders. In addition, small CBOs described an increase in recognition facilitated by their new affiliations with larger, more established organizations.

They brought … the hospital into the community. We now know more services that’s offered in the hospital as a result of being part of the partnership, and some of the benefits that were reaped from this partnership were the fact that now we have home visits for the elderly, and the sick, and infirm, and shut-in from [the hospital]. That was unheard of, but because of conversations, we were able to make that happen. (Stakeholder interview)

So, for elected officials, it’s raised our visibility. We had existed in the community for several years. We operated the Beacon Program for fifteen years. We operated at [school name] for eight years. We operated at [housing complex name] for four years. And we had never gotten any type of traction, in terms of funding, from our council person or our state officials. Where now, [our council person] allocated $100,000 for renovations right outside of the community center that needed to be done that hadn’t been done and another $100,000 for capital improvements just to get our internet up to speed. So, that’s helped. And I think CHVI has been a big part of that. And also, another thing I know, just in resources, getting our families able to be part of the [hospital] network, so that they can receive expedited appointments for checkups and physicals and things like that. That’s been helpful. (Stakeholder interview)

I would say that the core group of people who have been leading the interactions for the residents have been able to leverage a lot of city and neighborhood resources to participate – to let these people know that, “Yes, you’re a part of the city, and these are all the services that are available to you.” (Stakeholder interview)

#### Community Member Perspectives

The majority of community members participating in surveys and focus groups were not familiar with the Claremont Healthy Village Initiative or CHVI per se. However, many survey participants were aware of and participated in activities that fell under the CHVI umbrella. Two-thirds had participated in at least one CHVI-related activity, and many had participated in multiple activities (mean = 3 activities), including the annual family day held at one of the public housing developments, an annual health and wellness fair, and a Christmas event. Focus group participants also engaged in CHVI activities, including mental health first aid training, recreational activities, and educational programs, though in smaller numbers.

[The senior center is] really nice. We have a lot of activities and trips. You can learn the computer. There are people who sew and knit and crochet. There are a lot of things to do. I love it here. As soon as I hit sixty, I headed right here. (Focus group participant)

We had a program [in this housing complex] about a month ago, and it was a little program about bringing peace. We had an art program. Lovely time. It was very relaxing. Very engaging with the families that came. Children had a very nice time. (Focus group participant)

Focus group participants emphasized that additional programming was still needed, as well as better outreach, particularly for youth and older adults.

I should not have to go all the way to Harlem for my sons to play football. I should not have to go all the way uptown for my son to get in a basketball program. They need more programs. If we have more positive programs, the children will learn to get along with each other, because they find something that they have in common, so they can actually build instead of looking out the social media. (Focus group participant)

They felt that, without sufficient alternatives, youth end up on the internet or “hanging out,” creating an uncomfortable environment for other members of the community. Older adults were described as hesitant to leave their homes because of their fear of the youth.

It’s a benefit, but they’re just, you know, they’re not sure… I have to literally take people by their hand and say, “Come on, let me show you what I’m telling you.” I don’t know, how much more where we can make people, especially when you get older, but older people get afraid to come down. Six o’clock PM, “I’m not coming downstairs, and I can’t get back upstairs.” (Focus group participant)

### Impact on Health and Well-being

**Fig. 1:**
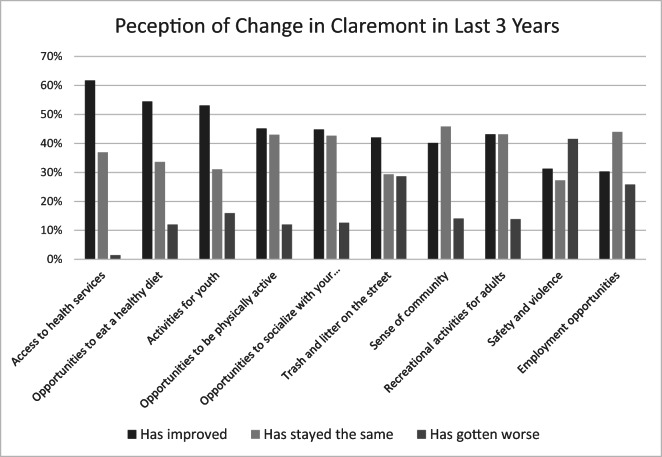
Perception of change in Claremont in last 3 years

Perceived changes to health services available to Claremont residents and changes to community factors that impact health were assessed through community member surveys (see Fig. 1 for survey findings), as well as key stakeholder interviews and focus groups. Across these methodologies, the most consistently positive responses focused on health care and nutrition. Sixty-two percent of survey participants reported increased access to health services in the years since CHVI was established.

Stakeholders and focus group participants described specific expansions in screening and management of disease, as well as facilitated access to health care services.

I feel more supported from the [hospital] aspect of it, because when I ask them for support, like if I need someone to come and do blood pressure at an event, family day, or we have the health fair initiative, Christmas extravaganza, very supportive. They will send a diabetes tester, blood pressure person, tons of information. Family day, the same thing. They’ll set up a table. They’ll be out there. (Focus group participant)

If we have a youth participant in our program and their grandmother or whomever needs medicine or needs some type of senior service, they're able to – we're able to pick up the phone [to the senior center] and say, "Hey, listen, one of ours needs something that you have, how can we make this work?" And then you throw in the hospital, where they have a whole department for physical health and mental health, that always comes up. And that's always an issue on some level. And so, having that resource, we've been able to pick up the phone or shoot [them] an email and say, you know, "This person needs a physical, this person's medicine isn't being carried or covered by the pharmacy anymore, and can your pharmacy give them something, you know, until this situation is resolved?" Or, “Can you get your health workers to do a presentation to our families on how to eat better, why to eat better?”… That is, I think the strength of their initiative. (Stakeholder interview)

A lot of people are more aware of different health and all that stuff. Such as asthma, high blood pressure, or prostate, breast cancer. You know, these people – they’ve been really helping these people out. Because a lot of them found out that they didn’t know that they had such things that were going on. (Stakeholder interview)

Increased opportunities to eat a healthy diet and increased activities for youth were reported by over half the survey respondents (54% and 53%, respectively); qualitative data suggest a range of opinions, with stakeholders reporting on expanded programing and focus group participants commonly emphasizing continuing needs, as alluded to in the previous section.

And so, that’s the way I see our impact is that we may not have a big impact with adults, but for [our organization] and our lens through CHVI, we’re really able to change and positively impact the next generation of Claremont residents to be more proactive and owning their own health, physical and mental health. (Stakeholder interview)

In the community member focus groups, violence and sanitation were considered very significant problems, generating extended discussions. As mentioned previously, several participants attributed violence in the community to youth who have insufficient options or are dealing with their anger in destructive ways. Roughly 40% of survey respondents felt that violence had worsened in the past 3 years, compared with 31% who felt it had improved and 27% who felt it had stayed the same. Survey respondents expressed more positive views on sanitation: 42% reported that sanitation had improved in recent years, compared with 30% who felt that it had gotten worse and 29% who felt it had stayed the same.

We’ve got twenty-one year-olds, twenty-two, a little younger than that; we got violence going on—robbing, mugging, stealing. It’s sad. That’s what’s going on now. That’s why you see so many cops, and they parking like check points. (Focus group participant)

So, you wanna find something get some boxing, get some martial arts, get some self-defense, because they’re angry and they [the youth] need to learn how to redirect their energy when they get angry instead of [using] guns and you know trying to kill each other. (Focus group participant)

If you’re here for so long, you’re not surprised by the rats [anymore], like you’re expecting a rat to come out. It doesn’t matter whether it’s daytime, nighttime, it’s just rats. (Focus group participant)

## Discussion

The Claremont Healthy Village Initiative promotes collaborative efforts that support programs and services to reduce inequities and address the broader determinants of health. The intentionally flexible structure provided by CHVI brings together a broad range of institutions and individuals working to improve community health and well-being. The local hospital and MCO serve as anchor organizations, facilitating communication and collaborations that include local tenant associations, community and senior centers, civic groups, public schools, cultural organizations, sports and recreation groups, and NYC governmental agencies. The anchor organizations do not claim or seek control, favoring a more diffuse decision-making process.

This evaluation of CHVI suggests positive outcomes at a structural level with respect to access to resources, referral networks and service coordination, empowerment of local community leaders, and expanded programming. These outcomes are consistent with those promoted within a community building approach, which focuses on developing synergies and building capacity of an entire system [12, 13]. They are aligned with the National Prevention Strategy [3] and Health in All Policies [14], in that health and wellness are promoted within CHVI in community-based settings and through organizations working in multiple sectors, including housing, education, and the arts. Understanding how multisector community partnerships such as CHVI impact organizations and individuals is increasingly important given the interrelated challenges of addressing health equity, building trust, and advocating for local needs [15].

At the individual level, outcomes were less evident, which is not surprising given the decades of disinvestment in the Claremont community. As one interviewee noted, “I’d say the realities of poverty still prevail, and undoing … systems that were built contrary to good health … takes a long time.” Due to these historical and persistent challenges, it may take many more years for an effort like CHVI to make a measurable impact on individual health, and the factors that impact it. That being said, it is promising that over half of community survey respondents perceived improvements in access to health services, opportunities to eat healthy, and activities for youth; all of which have been key components of CHVI programming efforts. Lesser progress was made with respect to violence and poorly maintained infrastructure. Although these were not priorities of CHVI at the outset, they are significant concerns of community members and of organizations that are part of the initiative. The slower progress in these areas may suggest a need for supplemental approaches, focused not only on community-based programs but on advocacy and policy change [1].

There were several challenges to conducting the evaluation, which were consistent with the literature on evaluation of coalitions [8] and reflective of expressed concerns regarding identification of best practices in community-wide approaches, more broadly [13]. The complexity and fluidity of the initiative presented challenges with respect to evaluation focus, which we tried to address through frequent bi-directional communication between the evaluation staff and the CHVI leads; the use of multiple methods and data sources; and flexibility, particularly in the early stages of the evaluation. Our initial protocol was modified as we came to better understand program activities and objectives, to ensure that findings could be used to improve, as well as inform [6]. There was significant variability in the level of engagement of the various partners, as well as the populations targeted and activities conducted. Although variability was consistent with CHVI’s naturalistic model, it led to challenges in understanding the value added of CHVI for some organizations and community members. A longer and larger evaluation could better assess outcomes, particularly at the individual level. It is also important to note that the evaluation described here began 4 years after CHVI was launched. The delay meant that CHVI had time to fully develop its approach; however, it also meant that the components of the evaluation described here (i.e., the primary data collection) had no real baseline. We attempted to address this challenge by asking respondents to reflect on the prior few years; however, we recognize that recall is likely to be imperfect. Despite these limitations, the evaluation had several strengths, including a mixed methods approach aimed at assessing CHVI at different levels—including processes for building community capacity. There was also a robust data collection effort; nearly 250 community members participated in surveys and focus groups, and 31 key stakeholder interviews were conducted. Several of the key stakeholders had been with CHVI from its inception, so they were able to recount its history in significant detail. These data provide a wealth of information about CHVI implementation, engagement, and community perceptions and should provide useful lessons to other coalitions.

## Conclusion

Multi-sector coalitions are commonly considered to be key mechanisms for addressing health equity and the broader determinants of health. Evaluation results suggest that CHVI, a multi-sector collaboration in an under-resourced neighborhood in the Bronx, has experienced success in terms of building collaborative and synergistic relationships among partner organizations, bringing resources and legitimacy to community organizations, and improving access to health promotion opportunities. These findings add to the evidence regarding the contribution of flexible multi-sectoral coalitions in addressing health inequities and increasing community capacity and connectivity in support of continued progress.

## References

[1] Woolf SH (2017). Progress in achieving health equity requires attention to root causes. Health Aff.

[2] Bor J, Cohen GH, Galea S (2017). Population health in an era of rising income inequality: USA, 1980-2015. Lancet..

[3] National Prevention Council (2011). National Prevention Strategy: America’s Plan for Better Health and Wellness.

[4] Clark NM, Lachance L, Doctor LJ, Gilmore L, Kelly C, Krieger J, Lara M, Meurer J, Friedman Milanovich A, Nicholas E, Rosenthal M, Stoll SC, Wilkin M (2010). Policy and system change and community coalitions : outcomes from allies against asthma. Am J Public Health.

[5] Tipirneni R, Vickery KD, Ehlinger EP (2015). Accountable communities for health: moving from providing accountable care to creating health. Ann Fam Med.

[6] Butterfoss FD (2007). Coalitions and partnerships in community health.

[7] Anderson L, Adeney K, Shinn C, Safranek S, Buckner-Brown J, Krause L. Community coalition-driven interventions to reduce health disparities among racial and ethnic minority populations (review ). *Cochrane Database Syst Rev*. 2015;6 10.1002/14651858.CD009905.pub2.www.cochranelibrary.com. 10.1002/14651858.CD009905.pub2 PMC10656573 26075988

[8] Granner ML, Sharpe PA (2004). Evaluating community coalition characteristics and functioning: a summary of measurement tools. Health Educ Res.

[9] Hinterland K, Naidoo M, King L, et al. Community health profiles 2018, Bronx Community District 3: Morrisania and Crotona. 2018. https://www1.nyc.gov/assets/doh/downloads/pdf/data/2018chp-bx3.pdf. Accessed 4 Apr 2019.

[10] Masseo P, Sanchez R, Realmuto L. *It takes a village: Claremont healthy village initiative builds body, mind and spirit*. Interdisciplinary association for population Health Sci (IAPHS). Clearfield, Utah. https://iaphs.org/takes-village-healthy-claremont-community-village-builds-body-mind-spirit/.

[11] Grootaert C, Narayan D, Jones VN, Woolcock M (2004). Measuring social capital: an integrated questionnaire.

[12] Walter CL, Hyde CA, Minkler M (2012). Community building practice: an expanded capital frameworks. Community Organizing and Community Building for Health and Welfare.

[13] Traynor B. Building community in place: limitations and promise. In: De Filippas J, Saegert S, editors. *Community Development Reader*. New York, NY: Routledge; 2012. chapter 23, p. 209–12.

[14] Rudolph L, Caplan J, Ben-Moshe K, Dillon L. *Health in all policies: a guide for state and local governments*. **Washington DC and Oakland CA: American Public Health Association and Public Health Institute**; 2013.

[15] Michener L, Aguilar-Gaxiola S, Alberti PM (2020). Engaging with communities — lessons (re) learned from COVID-19. Prev Chronic Dis.

